# Role of Oxidative Stress and Antioxidants in the Course of Atopic Dermatitis

**DOI:** 10.3390/ijms26094210

**Published:** 2025-04-29

**Authors:** Violeta Kvedariene, Monika Vaskovic, Justina B. Semyte

**Affiliations:** 1 Faculty of Medicine, Violeta Kvedariene Institute of Biomedical Sciences, LT-03101 Vilnius, Lithuania; 2 Faculty of Medicine, Vilnius University, LT-03101 Vilnius, Lithuania; vaskovic.monika@gmail.com (M.V.); justina.semyte@mf.stud.vu.lt (J.B.S.)

**Keywords:** oxidative stress, allergic dermatitis, atopic dermatitis, reactive oxygen species

## Abstract

Atopic dermatitis (AD) is one of the forms of allergic dermatitis and the most common chronic recurring inflammatory skin disease. In case of allergic dermatitis, oxidative stress (OS) promotes inflammation, disrupts the skin’s barrier function, and facilitates the penetration of allergens into the body. As a result, studying oxidative stress and its influence on the course and spread of these diseases is important in the search for new treatment strategies. This literature review aims to discover the effect of oxidative stress on the course of atopic dermatitis and review additional options for treatment. A comprehensive literature review was performed using the medical databases “PubMed” and the specialized search engine “Google Scholar” using the PICO model. Analyzed scientific articles were published from 2019 to 2024 in English. Of the 167 initial studies, 51 articles were included based on relevance, language, and release date. The other 116 articles were rejected due to incomplete publications and publications involving animals. Key biomarkers are associated with oxidative stress, including urinary 8-hydroxydeoxyguanosine (8-OHdG), malondialdehyde (MDA), glutathione, and glutathione disulfide, and they correlate directly with the severity of atopic dermatitis. This research emphasizes that antioxidants, such as vitamins, sun protection, coenzyme Q10, a balanced diet, melatonin, flavonoids, and NB-UVB therapy may have a positive impact on the pathogenesis and progression of atopic dermatitis.

## 1. Introduction

**Figure 1:**
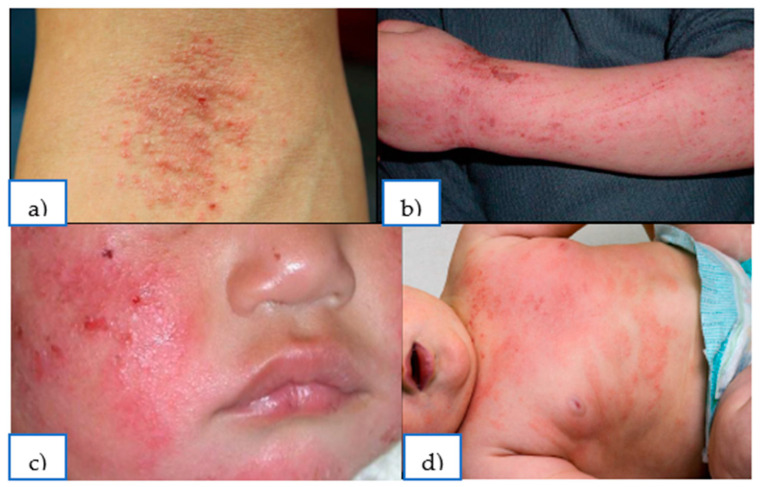
Clinical manifestations of atopic dermatitis: (**a**) flexural erythematous patches and papules; (**b**) excoriated, lichenified lesions; and (**c**) infantile erythematous lesions on the cheeks and (**d**) trunk.

Atopic dermatitis (AD) has a great influence on the health and quality of life of people from early childhood to old age. Atopic dermatitis is a chronic, allergic, inflammatory skin disease characterized by intense itching, eczematous skin lesions, and a relapsing course [1]. Oxidative stress (OS), in comparison with many other pathophysiological mechanisms, is characterized by being a very important factor that increases the severity of these diseases and their progression. Oxidative stress is defined as the formation of oxidants in the cells of the human body and the disruption of their accumulation and neutralization, leading to the structural components of the cell being damaged due to the destruction of proteins and changes in the structures of the cell membrane and the nucleus [2]. In the case of allergic dermatitis, oxidative stress promotes inflammation, disrupts the barrier function of the skin, and facilitates the penetration of allergens into the body [3]. Thus, symptoms may be provoked and present as skin itching, dryness, lichenifications, and eczema-like skin lesions with erythematous patches and papules (Figure 1). Antioxidants neutralize the effects of oxidative stress, and they can be used to alleviate clinical symptoms of atopic dermatitis and measure the impact of oxidative stress on the skin [2].

The incidence of atopic dermatitis is increasing, but the number of effective treatment methods is limited. As a result, studying oxidative stress and its influence on the course and spread of these diseases is important in the search for new treatment strategies.

## 2. Methodology

The search and analysis of the scientific literature were carried out using the medical database “PubMed” (Medline) and a specialized search in “Google Scholar”. Keywords and their combinations were used in the search: “oxidative stress”, “allergic dermatitis”, “atopic dermatitis”, and “dermatitis”.

### 2.1. Inclusion Criteria

Scientific literature in which subjects were diagnosed with allergic dermatitis.

The scientific literature presents the influence of oxidative stress on the course of allergic dermatitis.

Research that examines biomarkers of oxidative stress on the severity of the disease.

Clinical, randomized, case control, prospective, or retrospective observational studies and systematic reviews.

Scientific literature published in English.

Research was published no more than 5 years ago.

### 2.2. Exclusion Criteria

Clinical studies on other diseases influenced by oxidative stress (e.g., diabetes, cardiovascular diseases).

Experimental research.

Studies that do not present the influence of oxidative stress on the course of allergic dermatitis.

Studies published in languages other than English.

### 2.3. Detalis of Analysis and Interpretation

Using the keywords mentioned above, 167 articles were identified in the electronic databases. In the first selection stage, articles were chosen based on whether titles or abstracts met all of the criteria for inclusion. During the second stage, the full text of the publication was read, and articles that met the criteria for exclusion were rejected. The remaining publications were included in the literature review.

### 2.4. Potential Biases and Limitations

Despite the rigorous article selection process, there may still be potential biases and limitations in this literature review. The studies included articles published only in the English language and limited to the last 5 years of publication. This may have excluded relevant studies that could provide additional important data. Additionally, the literature included published articles that include various methodologies, diagnostic criteria, and biomarkers. As a result, this may complicate the data synthesis process and affect the understanding of how precisely oxidative stress influences the course of atopic dermatitis. Future studies are needed to further explore the consequences of oxidative stress in atopic dermatitis.

## 3. Features of Atopic Dermatitis

Atopic dermatitis (AD) is the most common chronic recurring inflammatory skin disease, affecting up to 25% of children and about 5% of adults worldwide [4]. The symptoms of AD include skin itching, dryness, and eczema-like skin lesions, with erythematous patches and papules that can become indistinct and lichenified with serous exudate over the course of the disease. AD symptoms are categorized as acute, subacute, and chronic. Acute symptoms include edematous, erythematous papules and plaques, vesicles, and crusts; subacute symptoms include erythema and scaling; and chronic symptoms consist of thick plaques with lichenification and scaling [5].

AD symptoms depend on the age group. In infants, edematous papules and plaques appear on the scalp, face, and surfaces of extending limbs; in some cases, vesicles may also occur. In older children, rashes typically appear on the flexural surfaces. In adults, AD is most common on the flexural surfaces of the body, such as the inner areas of the elbows, knees, neck, and face, presenting as dry, scaly, and thickened skin [6].

AD is a genetically predisposed disease, and its prevalence is influenced by environmental factors, skin barrier defects, immune system disorders, and hypersensitivity. Living in an industrialized area and in a dry climate are among the most studied factors that worsen the course of the disease [7].

A defect in the skin barrier can occur due to mutations in the *filaggrin* gene. In the outer layer of the epidermis, there is a lack of functional filaggrin peptides, which leads to increased skin permeability. This allows harmful external factors to penetrate the atopic skin more easily. Damaged keratinocytes produce alarmins, such as interleukin (IL)-33, IL-25, and thymic stromal lymphopoietin (TSLP). These alarmins activate dendritic cells and type 2 innate lymphoid cells (ILC2), which produce IL-5 and IL-13, activating eosinophils and Th2 cells [8].

Th2 and other subgroups of T cells contribute to the pathogenesis of AD. In the acute and chronic stages of AD, a distorted response of Th2 is observed, leading to an increase in the activity of IL-4, IL-5, IL-13, and IL-31 [9]. These type 2 cytokines inhibit barrier proteins and lipids, contributing to barrier dysfunction. They also activate immune cells, such as eosinophils and mast cells, and facilitate changes in the isotype of B cells, promoting the production of immunoglobulin E (IgE) at elevated levels. High levels of IgE are associated with allergic reactions and contribute to the worsening of symptoms, including inflammation and itching [10].

It has been observed that AD, due its notable inflammatory component, is affected by oxidative stress. Therefore, there has been growing discussion regarding the impact of OS, as it could improve our understanding of the development and maintenance and treatment options for atopic dermatitis [11].

## 4. Mechanism of Oxidative Stress

Oxidative stress (OS) is defined as the formation of free radicals in the cells of the human body and the disruption of the accumulation and neutralization of these compounds [12]. Free radicals, including reactive oxygen species (ROS) and forms of nitrogen oxides, are induced by exogenous and endogenous factors, such as physiological metabolic products of cells that activate immune cells, including neutrophils, macrophages, and T lymphocytes. Over the course of pathological processes, more free radicals are formed than neutralized, and this leads to the development of OS [13].

With an acute change in the level of oxygen in the body, during processes like hyperoxia or hypoxia, ROS are formed. Most often, ROS are a product of the reduction of one oxygen atom. The most common and highly toxic form of ROS, the superoxide anion (O_2_•−), is formed as a by-product of mitochondrial respiration in the electron transport chain when oxygen is reduced by a single electron in the mitochondrial respiratory chain [14]. Under the influence of the enzyme superoxide dismutase, two superoxide anions combine to form hydrogen peroxide (H_2_O_2_), which is less reactive. When hydrogen peroxide is reduced by one electron, a hydroxyl radical (•OH) is formed, which is a highly damaging ROS. In addition, it is formed during the Haber–Weiss or Fenton reactions, and it is characterized by a very short half-life (10^−9^ s) [15]. The idea that a reactive hydroxyl radical can be formed by the interaction of superoxide and hydrogen peroxide (O_2_•− + H_2_O_2_ → •OH + O_2_ + H_2_O) was proposed back in 1934 in Professor Haber’s scientific work. After discovering that the superoxide anion is a physiological cell metabolite, it was quickly recognized that the Haber–Weiss reaction is the cause of the formation of toxic radicals [16]. Meanwhile, the Fenton reaction, named after the observations of chemist H.J.H. Fenton in 1880, shows that a hydroxyl radical (Fe^2+^ + H_2_O_2_ → Fe^3+^ + •OH + H_2_O) is formed in a mixture of acidified iron and H_2_O_2_ [17].

The formation of ROS is influenced by exogenous and endogenous factors. External factors include ultraviolet (UV) radiation, tobacco smoke, pollutants, stress, infections, and allergens, while internal factors depend on mitochondrial activity, enzymes, such as nicotinamide adenine dinucleotide phosphate (NADPH) oxidase, and various cell interactions [18]. Elevated concentrations of ROS can cause nonspecific damage to macromolecular targets. They can cause irreversible damage to DNA, lipids, enzymes, and proteins, as ROS oxidizes and modifies these macromolecular targets and prevents them from performing their functions [18]. ROS are extremely unstable, and, in order to stabilize, they absorb electrons from nearby macromolecules, thereby causing damage, which can ultimately lead to apoptosis or necrosis [19].

## 5. Classification of Antioxidants

**Figure 2:**
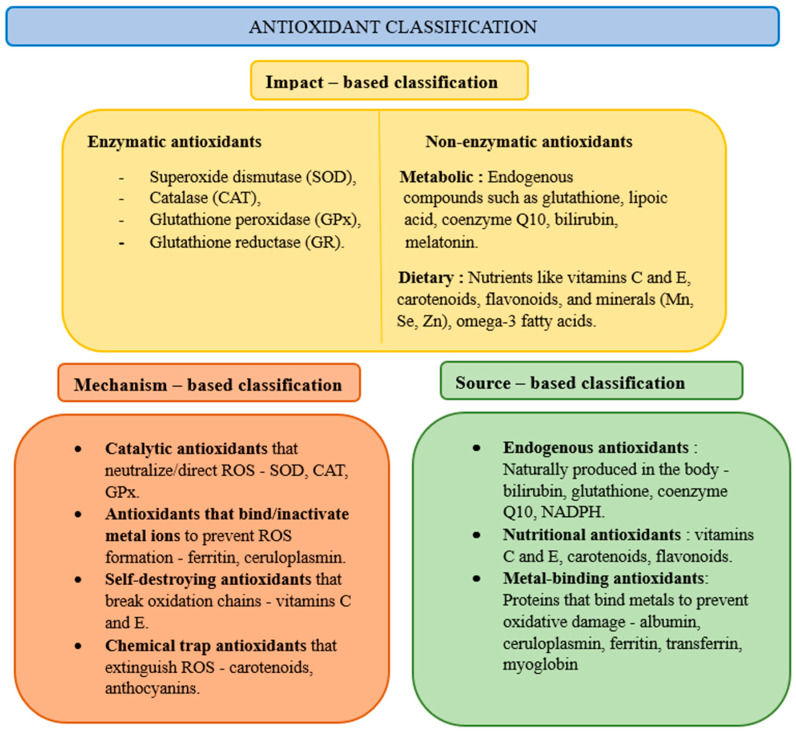
Classification of antioxidants according ref. [22].

Biological antioxidant protection systems exist in cells. Antioxidants can be divided into primary (prevention against the formation of ROS), secondary (ROS inactivators), and tertiary (restoring oxidized molecules), taking into account their effects, enzymatic and non-enzymatic, their mechanism of action, and their source of occurrence (Figure 2). The most important enzymatic antioxidants are superoxide dismutase, catalase, glutathione peroxidase, and glutathione reductase, while non-enzymatic ones include glutathione, vitamin C (ascorbic acid), vitamin E (α-tocopherol), polyphenols, coenzyme Q10, melatonin, and carotenoids [20]. Superoxide dismutase enzymes catalyze the conversion of O_2_•− into H_2_O_2_. Then, the resulting H_2_O_2_ is reduced by glutathione peroxidase enzymes, using glutathione as a cofactor and forming H_2_O and oxidized glutathione. Glutathione reductase is involved in the regeneration of oxidized glutathione. With the activation of oxidative stress, the enzymatic and non-enzymatic antioxidant responses increase, enhancing their activity to neutralize oxidative stress [21].

The direct effect of reactive oxygen species (ROS) can be assessed by measuring the levels of biomarkers. Urinary 8-hydroxy-2-deoxyguanosine (8-OHdG) is a biomarker for oxidative DNA damage and restoration. Lipid peroxidation biomarkers include malondialdehyde (MDA), nitric oxide (NO), and 4-hydroxy-2-nonenal (HNE) [2]. Malonialdehyde is a serum biomarker typically measured using thiobarbituratic acid and detected through spectrophotometry [23]. Nitric oxide is usually tested in urine and measured by determining the cumulative amount of urinary nitrite/nitrate (NO_x_^–^) molecules. HNE biomarkers can be detected in various tissues. Scientific research has shown a substantial increase in different types of clinical samples, such as skin, ocular brush cytology, and exhaled breath condensates, using different measurement methods [2]. Lastly, serum markers, such as advanced oxidation protein products (AOPPs) and advanced glycation end products (AGEs), are formed as a result of protein oxidation [2]. These molecules are measured using spectrofluorimetry and spectrophotometry, respectively [24].

Taking into account all of the biomarkers mentioned above, nitric oxide, indirectly measured based on nitrite/nitrate levels, is the only marker with more established clinical applications (e.g., in conditions like diabetes [25] or liver cirrhosis [26]). However, as research progresses, other biomarkers may also gain more attention and diagnostic accuracy in clinical practice for patients with atopic dermatitis. The disruption of the balance between accumulation and neutralization of ROS, which causes oxidative stress, affects the development of allergic dermatitis and exacerbates its course [3].

## 6. The Effect of Oxidative Stress on the Course of Atopic Dermatitis

Oxidative stress is important in the pathogenesis of atopic dermatitis, and it has a significant influence on its course. It can damage keratinocytes, promote skin inflammation, and reduce the skin’s barrier function. Measurement of oxidative stress is possible by identifying peripheral markers that have prognostic significance for the severity, progression, and response to treatment of the disease. Oxidative stress is assessed using various biomarkers in urine and blood serum [3].

Oxidative stress provokes a strong exacerbation of allergic contact dermatitis. This is evidenced by the very high ratio of glutathione disulfide (GSSG) to glutathione (GSH) compared to values observed after dermatitis has resolved. The earliest phase of lipid peroxidation involves the reduction of hydrogen, during which dehydration of alkanes leads to the formation of alkadienes—instead of double C=C bonds, single C-C bonds are formed. During this process, conjugates of dienes are formed, which are markers of oxidative stress. In order for the conjugates of dienes to be neutralized, glutathione oxidizes to glutathione disulfide. A decrease in the amount of GSH and an increase in GSSG are indicators of oxidative stress and the overall antioxidant capacity of cells. Oxidative stress causes inflammation, which is manifested by erythema, swelling, itching, and the formation of blisters [27].

In patients with atopic dermatitis (AD), there is a sharp change in the levels of urinary 8-hydroxydeoxyguanosine, nitrites, nitrates, and selenium, with significant increases observed during exacerbations of the disease or hospitalization. These markers correlate directly with the severity of the disease [28]. Urinary 8-OHdG is an oxidized nucleoside and a biomarker of oxidative DNA damage. When DNA is damaged and subsequently repaired, 8-OHdG is excreted in urine, so its levels can reflect the extent of oxidative DNA damage. Additionally, higher urinary 8-OHdG levels in AD patients are associated with a greater risk of developing asthma [29].

The most vulnerable biomolecules in oxidative stress are lipids, especially polyunsaturated fatty acids, which have a high number of double carbon–carbon (C=C) bonds. ROS, such as the hydroxyl radical, bind to hydrogen from polyunsaturated fatty acids, resulting in the formation of unstable lipid radicals (L•). Later, when reacting with oxygen, lipid peroxide radicals (LOO•) are formed, which bind to another hydrogen from another lipid molecule, forming more stable compounds—lipid hydroperoxides (LOOH). This process, called lipid peroxidation, can lead to the decomposition of both lipid hydroperoxides and lipid peroxide radicals, forming secondary products [30].

Malondialdehyde (MDA) is the main and most widely studied compound formed during lipid peroxidation, known for its mutagenic and toxic effects [23]. MDA is a biomarker of oxidative stress that plays an important role in diagnosing atopic dermatitis and monitoring the disease’s course. A case-control study conducted in 2016 showed that patients with AD have higher levels of the lipid peroxidation marker malondialdehyde and lower levels of vitamins A, C, and E, which have antioxidant functions, compared to the control group [11].

Lipid oxidation causes damage to DNA, cell enzymes, and cell membrane structures, affecting the keratinocytes of the epidermis. These intracellular changes are histomorphologically manifested as edema of the epidermis and damage to the stratum corneum. Some of the most important lipids are ceramides, which are responsible for maintaining the skin’s barrier function. Ceramides are composed of sphingosine and fatty acids and synthesized in the stratum corneum during keratinization. The main function of the epidermal barrier is to restrict the entry of allergens and infectious agents into the body and to prevent transepidermal water loss. Studies have shown that there are significantly fewer proteins associated with skin barrier function in areas of skin barrier damage, such as filaggrin-2, corneodesmosin, desmoglein-1, democolin-14, and transglutaminase-3 [31].

Studies have also proven that oxidative stress (OS), initiated by external pollutants, directly compromises the skin barrier. In skin biopsies of atopic dermatitis (AD) patients, it was observed that the content of dinitrophenylhydrazine (DNP), a marker of oxidative protein damage, is elevated in AD lesions and correlates with the severity of AD. It has also been noted that DNP is more intensely formed in the superficial stratum corneum layer than in deeper layers, suggesting that oxidative damage may be associated with the effects of environmental oxidants. Thus, an increase in the amount of reactive oxygen species (ROS) due to environmental pollutants and ultraviolet rays can cause oxidative protein damage in the epidermis, leading to impaired skin barrier function and exacerbation of AD [32].

The sources of oxidative stress in patients with AD can be environmental, physical, and psychological. Various air pollutants, such as tobacco smoke, volatile organic compounds, and nitrogen dioxide, are known risk factors for AD and can aggravate the course of the disease. Aryl hydrocarbon receptors (AhR) and aryl hydrocarbon receptor nuclear translocator (ARNT) have been shown to play an important role in keratinocytes. AhR ligation causes not only OS but also a ligand-dependent antioxidant response. Environmental pollutants, such as cigarette smoke, bind to AhR, causing ROS production, DNA damage, and the production of inflammatory cytokines, resulting in impaired skin barrier function and immune dysregulation, thus leading to skin inflammation [33].

Tobacco smoke is a source of ROS in the body, and it is associated with the development of AD. Contact dermatitis in several cases could be associated with atopic dermatitis. During smoking, molecular oxygen in the lungs is reduced to a superoxide anion, which is then reduced to a hydroxyl radical, causing inflammation and disrupting the barrier function of the skin. Additionally, the metals contained in tobacco smoke are oxidative stress factors, as well. Traces of heavy metals found in tobacco leaves, such as chromium, nickel, iron, and copper, are known contact allergic dermatitis irritants and involved in the Fenton reaction, thereby contributing to OS [34].

Ultraviolet (UV) rays are another factor in the formation of ROS in the skin. Ultraviolet radiation consists of three components: UVA (wavelength between 320 nm and 400 nm), UVB (wavelength between 280 nm and 320 nm), and UVC (wavelength between 100 nm and 280 nm). UVC radiation is believed to be the most dangerous type, but, in contrast to UVA and UVB, it is fully absorbed by the ozone layer, which significantly reduces its effect on the skin [35]. ROS, such as H_2_O_2_ and OH•, form on the skin after just 15 min of UV exposure, and their levels continue to increase for the first 60 min of UV radiation [32].

However, purposefully dosed rays as phototherapy treatment can be used in the treatment of atopic dermatitis. UV therapy utilizes different wavelengths of UV rays. UVA therapy is further divided into ultraviolet 1 therapy (UVA1, 340–400 nm) and ultraviolet 2 therapy (UVA2, 320–340 nm), while UVB is split into broadband UVB (BB-UVB, 90–320 nm) and narrowband UVB (NB-UVB, 311–313 nm) [36]. Another treatment option is PUVA (psoralen and long-wave ultraviolet radiation) photochemotherapy, which combines oral or topical administration of psoralens and UVA radiation. Researchers have found that high-dose UVA1 and medium-dose UVA1 cold light offer the best long-term effects for treating AD [37]. However, NB-UVB therapy is recognized to be more clinically tolerable with fewer side effects compared to other UV phototherapies [36,38]. Therefore, when selecting a UV therapy, it is important to consider clinical characteristics of atopic dermatitis and individual patient health factors.

ROS is formed when photons absorb endogenous photosensitizer molecules, such as cytochromes, riboflavin, heme, and porphyrin. The excited photosensitizer subsequently reacts with oxygen, resulting in the formation of a superoxide anion. Superoxide dismutase converts O_2_- to H_2_O_2_, which easily passes through cell membranes along with Fe (II) and Cu (II) and promotes the formation of a hydroxyl radical [15]. In this way, the formation of ROS, stimulated by UV radiation, leads to oxidative protein damage and lipid peroxidation, which leads to impaired skin barrier function and exacerbation of AD [3].

Psychological stress is another well-known cause of the OS, causing skin barrier dysfunction, and it is a common factor in the exacerbation of AD [39]. Psychological stress leads to an increase in the level of endogenous corticosteroids, which disrupts not only the barrier function of the skin but also the antimicrobial protection of the epidermis of the skin. Due to poor sleep patterns, psychosocial burdens, and poor quality of life for many AD patients, a link has been established between AD and depression. AD patients have a 59 percent higher chance of developing depression, which may be associated with neuro-inflammatory pathways [40].

## 7. Research on New Biomarkers of Oxidative Stress in Atopic Dermatitis

In practice, new biomarkers, such as bilirubin, carbonyl molecules, and modified amino acids, are being identified. However, not all of these biomarkers are sufficiently studied.

Bilirubin has strong antioxidant and protective effects. An imbalance of reduction and oxidation, along with an increase in the level of reactive oxygen species (ROS), leads to the oxidation of bilirubin. The oxidative metabolites of bilirubin are called urinary biopyrrins (UBP), which are rapidly excreted in urine. Increased oxidation of bilirubin correlates with elevated levels of urinary biopyrins, making it a potential biomarker for oxidative stress (OS). The amount of UBP increases with stress, including psychological and social stress, as well as in atopic dermatitis (AD). It is likely that bilirubin is oxidized to dipyrrole–monopyrrole–aldehyde in patients with AD; therefore, to accurately assess the severity of AD, it is important to develop tools to determine the levels of dipyrrole–monopyrrole–aldehyde in the body [41].

An excess of ROS formed as a result of OS reacts with proteins and causes oxidative damage. Advanced oxidation protein products are recognized as new OS biomarkers formed when proteins interact with oxidants. The amount of carbonyl moieties produced during this process increases during AD and correlates with the severity of the disease [42]. Current studies suggest that modified amino acids, such as 6-nitrotryptophan and nitrotyrosine, could be potential biomarkers, but existing studies are insufficient to establish this relationship [43].

## 8. Suppression of Symptoms of Atopic Dermatitis Caused by Oxidative Stress

Atopic dermatitis can be treated various ways (for example, topical corticosteroids, antihistamines, and phototherapy); however, very few studies examine how to treat the condition caused by oxidative stress (OS).

Taking ascorbic acid and α-tocopherol supplements reduces the condition caused by OS. During the remission of contact allergic dermatitis (CAD), the glutathione disulfide/glutathione ratio is significantly reduced, but it does not reach normal levels, indicating chronic OS. Ascorbic acid and α-tocopherol are essential components of the non-enzymatic antioxidant system. The levels of these vitamins in the skin decrease under the influence of UV radiation, which generates free radicals [44]. Vitamin E is the primary lipophilic antioxidant in plasma lipoproteins and cell membranes. It stabilizes the skin’s barrier lipids and prevents peroxidation [45]. Glutathione (GSH) and vitamin C are involved in synthesizing α-tocopherol from α-tocopherol radicals. Vitamin C supplements increase the level of vitamin E in the blood, and the latter helps maintain and elevate GSH levels in keratinocytes by promoting the synthesis of γ-glutamyl-cysteine. The use of vitamin E and C supplements promotes an increase in GSH levels and normalizes the GSSG/GSH ratio, thereby reducing OS and its determinant condition in contact allergic dermatitis [46].

An essential preventive and therapeutic measure to protect against oxidative stress is photoprotection from exposure to sunlight on the surface of the skin. Sun Protection Factor (SPF) sunscreens are an effective way to protect oneself from harmful UV rays. It is important to choose an SPF filter that protects against both UVB and UVA rays. UVA rays can penetrate deeper into the skin and cause oxidative damage, which, in the long run, affects the development of allergic dermatitis [47].

Coenzyme Q10 is an antioxidant produced naturally in the body. Due to exposure to ultraviolet rays and other factors that cause OS, the level of coenzyme Q10 in the body can significantly decrease. Coenzyme Q10, also called ubiquinone, is a fat-soluble, vitamin-like benzoquinone compound that is endogenously synthesized in the human body from tyrosine. It is a strong lipophilic antioxidant capable of neutralizing free radicals and regenerating the reduced form of vitamin E. Coenzyme Q10 also inhibits lipid peroxidation in biological membranes and protects mitochondrial proteins and DNA from OS. The daily dose of antioxidant supplements depends on the indications. Typically, people without medical conditions are offered a daily dose of 30–100 mg, and for people with chronic diseases, such as AD, the daily dose can reach up to 60–1200 mg [48].

Studies show that the development of OS is influenced by an unbalanced diet, including, for example, low consumption of fruits and vegetables and high consumption of sugar and processed foods. Intake of vitamin E, beta-carotene, folic acid, and iron through food and dietary supplements is associated with a lower risk of AD [49].

Melatonin is a hormone that is mainly produced by the pineal gland. It exhibits cytoprotective, immunomodulatory, and anti-apoptotic effects [50]. This hormone is also one of the most powerful natural antioxidants, directly acting to neutralize free radicals. Melatonin stimulates important antioxidant enzymes, such as superoxide dismutase, glutathione peroxidase, and glutathione reductase, which protect cell membranes from lipid peroxidation [51]. In addition, melatonin neutralizes ROS formed due to ultraviolet rays. In patients with allergic dermatitis, melatonin supplements reduce inflammatory cytokines, lipid peroxides, and ROS levels [52].

Flavonoids are low-molecular-weight plant metabolites with a wide range of effects, including antioxidant, antibacterial, antiviral, anti-inflammatory, and antiallergic effects [53]. The antioxidant effect of flavonoids is based on the ability of aromatic hydroxyl groups to transfer the H^+^ ion to free radicals, thus forming a stable flavonoid radical [54]. Quercetin is a polyphenolic flavonoid found in certain foods. It inhibits the release of histamine from basophils and mast cells and inhibits the emission of pro-inflammatory cytokines, such as IL-4 and IL-13 [55]. In addition, quercetin increases the content of enzymes with antioxidant effects, such as superoxide dismutase, catalase, and glutathione peroxidase. A low concentration of flavonoids can be achieved by consuming flavonoid-rich foods, such as leafy vegetables, onions, apples, berries, and citrus fruits. It is important to note that a high content of flavonoids can also have a detrimental effect due to their pharmacological properties. Some flavonoids can act as pro-oxidants, producing ROS. In order to avoid excessive formation of ROS, the doses of flavonoids should not be higher than those that are absorbed with a vegetarian diet. Studies have observed a decrease in eosinophil counts and acute AD after a two-month vegetarian diet that included foods rich in flavonoids [56].

## 9. Conclusions

Oxidative stress has become a current topic of modern scientific research exploring and discovering new biomarkers that affect the course of allergic dermatitis. It plays an important role in the pathogenesis of atopic dermatitis and affects the severity and progression of the disease. Exogenous and endogenous factors play a significant role in the formation of reactive oxygen species (ROS). Elevated levels of ROS can cause nonspecific damage to DNA, lipids, enzymes, and proteins. ROS can be classified in various ways, but the simplest categorization is based on their origin.

Key enzymatic antioxidants include superoxide dismutase, catalase, glutathione peroxidase, and glutathione reductase. Non-enzymatic antioxidants are equally important and consist of glutathione, vitamin C (ascorbic acid), vitamin E (α-tocopherol), polyphenols, coenzyme Q10, melatonin, and carotenoids. Conversely, reactive oxygen species oxidize and modify macromolecular targets, hindering their functionality.

Furthermore, incorporating antioxidants into clinical practice could reduce the symptoms of severe atopic dermatitis by modifying oxidative stress mechanisms involved in AD pathogenesis. In terms of flavonoid complex dietary supplements, most non-enzymatic antioxidants can be recommended in addition to conventional treatments for atopic dermatitis to achieve better clinical outcomes. For AD patients with sleep impairment, melatonin supplements may help reduce inflammatory cytokines, lipid peroxides, and ROS levels, thereby improving both sleep quality and skin condition.

Phototherapy treatment for AD is recognized to improve skin conditions. With regard to long-term effects, high-dose UVA1 and medium-dose UVA1 cold light are found to be treatment options for atopic dermatitis. However, NB-UVB therapy is shown to be more clinically tolerable with fewer side effects. Thus, phototherapy is an additional AD treatment for dermatologists, given the clinical presentation and individual patient factors, to achieve the best possible outcome with a minimal side effects.

In addition, external factors and lifestyle choices, such as a hectic and stressful lifestyle, affect the formation of a large number of free radicals. Smoking increases the amount of reactive oxygen species, which leads to inflammation and disruption of the skin barrier, contributing to acute atopic dermatitis. Avoidance of smoking and stress is very important for the prevention of atopic dermatitis exacerbation. Quality sleep not only stimulates the production of melatonin, which is a strong antioxidant, but also alleviates psychological stress. Eating a balanced diet rich in antioxidants and avoiding environmental pollutants, noncontrolled ultraviolet rays, and psychological stress could alleviate the course of atopic dermatitis and reduce exacerbations.

To better understand the pathogenesis of atopic dermatitis (AD), it is essential to identify its biomarkers. The impact of oxidative stress on the progression of AD can be assessed through various biomarkers. These include urinary 8-hydroxy-2-deoxyguanosine, malondialdehyde, nitric oxide, and 4-hydroxy-2-nonenal, as well as serum markers like advanced oxidation protein products.

To accurately evaluate the severity of AD, it is crucial to develop methods for measuring dipyrrole–mono pyrrole–aldehyde levels in the body and to analyze modified amino acids. Additionally, 6-nitrotryptophan and nitrotyrosine may serve as potential biomarkers, although further research is needed to validate this hypothesis. Currently, these tests are only conducted in research settings. As research advances, it is likely that more oxidative stress biomarkers will become available in clinical practice, enhancing diagnostic accuracy for atopic dermatitis. Despite recent scientific progress, further studies are required to explore new oxidative stress biomarkers associated with severe atopic dermatitis and to investigate the role of antioxidants in the routine treatment of severe atopic conditions.
